# Fibroblast Growth Factor 21 and the Adaptive Response to Nutritional Challenges

**DOI:** 10.3390/ijms20194692

**Published:** 2019-09-21

**Authors:** Úrsula Martínez-Garza, Daniel Torres-Oteros, Alex Yarritu-Gallego, Pedro F. Marrero, Diego Haro, Joana Relat

**Affiliations:** 1 Department of Nutrition, Food Sciences and Gastronomy, School of Pharmacy and Food Sciences, Food Torribera Campus, University of Barcelona, E-08921 Santa Coloma de Gramenet, Spain; ursula-mtz@hotmail.com (U.M.-G.); danytoot@hotmail.com (D.T.-O.); aleyaga@gmail.com (A.Y.-G.); pedromarrero@ub.edu (P.F.M.); 2 Institute of Nutrition and Food Safety of the University of Barcelona (INSA-UB), E-08921 Santa Coloma de Gramenet, Spain; 3 Institute of Biomedicine of the University of Barcelona (IBUB), E-08028 Barcelona, Spain; 4 CIBER Physiopathology of Obesity and Nutrition (CIBER-OBN), Instituto de Salud Carlos III, E-28029 Madrid, Spain

**Keywords:** carbohydrates, fat, fibroblast growth factor 21, gene expression, liver, metabolic homeostasis, nutritional challenges, protein, signal transduction

## Abstract

The Fibroblast Growth Factor 21 (FGF21) is considered an attractive therapeutic target for obesity and obesity-related disorders due to its beneficial effects in lipid and carbohydrate metabolism. FGF21 response is essential under stressful conditions and its metabolic effects depend on the inducer factor or stress condition. FGF21 seems to be the key signal which communicates and coordinates the metabolic response to reverse different nutritional stresses and restores the metabolic homeostasis. This review is focused on describing individually the FGF21-dependent metabolic response activated by some of the most common nutritional challenges, the signal pathways triggering this response, and the impact of this response on global homeostasis. We consider that this is essential knowledge to identify the potential role of FGF21 in the onset and progression of some of the most prevalent metabolic pathologies and to understand the potential of FGF21 as a target for these diseases. After this review, we conclude that more research is needed to understand the mechanisms underlying the role of FGF21 in macronutrient preference and food intake behavior, but also in β-klotho regulation and the activity of the fibroblast activation protein (FAP) to uncover its therapeutic potential as a way to increase the FGF21 signaling.

## 1. Introduction

The Fibroblast Growth Factor (FGF) family comprises 23 structurally related proteins divided into seven sub-families according to their phylogenetic similarity. They show pleiotropic effects on development, organogenesis, cell growth, differentiation, and survival, but also on metabolism [1]. With the exception of the FGF11 subclass members, named intracellular or nuclear FGFs, the FGFs act as autocrine, paracrine, or endocrine factors and signal by binding to a fibroblast growth factor tyrosine kinase receptor (FGFR). Four different genes encode for seven isoforms of FGFRs: 1b, 1c, 2b, 2c, 3b, 3c, and 4.

### 1.1. The Endocrine FGFs

The members of the FGF19 sub-family of FGFs, also known as endocrine FGFs (eFGFs), show an atypical structure. They lack the extracellular heparin-binding domain, which means a lower heparin-binding affinity and no capacity for being retained in the extracellular matrix, thus conferring on them the ability to enter the systemic circulation [2]. The eFGFs require the dimerization of an FGFR (FGFR1c, 2c, 3c, or 4) with a co-receptor to activate their signal transduction pathways [2,3]. This co-receptor is a klotho protein, alpha klotho for FGF23, and beta-klotho (KLB) for FGF19 and 21. Downstream of the FGFR-KLB receptors, the intracellular cascade goes through the phosphorylation of FGFR substrate 2α (FRS2α) and the activation of Ras-MAPKs and PI3K-Akt kinases [3,4,5].

Globally, the eFGFs play an important role in maintaining metabolic homeostasis [6,7]. FGF23 participates in the crosstalk between the bone and kidney, the intestine, and the parathyroid gland to maintain the body mineral balance, vitamin D3 homeostasis, and, finally, bone health [6]. On the other hand, FGF19 (FGF15 in rodents) and FGF21 are involved in the maintenance of body weight and metabolic homeostasis by regulating glucose and lipid metabolism [6,7,8]. 

### 1.2. FGF21 and Metabolic Homeostasis 

FGF21 was first described in 2000 by Nishimura et al. [9] and is considered an attractive therapeutic target to treat obesity and obesity-related metabolic disorders due to its beneficial effects in lipid and carbohydrate metabolism [10,11,12,13]. Some of the described FGF21 analogues or activators are classified as anti-obesity and antidiabetic molecules that improve insulin sensitivity, ameliorate hepatosteatosis, and promote weight loss [14,15,16,17,18]. As has been mentioned before, FGF21 signaling requires KLB to activate FGFRs, and the co-expression of these two receptors determines the sensitivity of a tissue or organ to FGF21 [5,19,20,21]. 

FGF21 is mainly produced by the liver, but is also secreted in skeletal muscle, white adipose tissue (WAT), brown adipose tissue (BAT), the intestine, heart, kidneys, and pancreas, and it is defined as a stress-responsive hormone [22]. Besides its action as an endocrine FGF, FGF21 also acts in a paracrine and autocrine way. It has been described that the FGF21 produced by WAT acts mainly in an autocrine way by activating the peroxisome proliferator-activated receptor-γ (PPARγ) [23,24]. By contrast, the FGF21 secreted by the liver, skeletal muscle, heart, or BAT can exert its effects through endocrine, paracrine, and autocrine signaling [25,26,27]. The effects of FGF21 are subtle under normal conditions but increase significantly under metabolic, oxidative, nutritional, hormonal, or environmental challenges, reinforcing its key role in restoring metabolic homeostasis [5,25,28,29,30]. FGF21 response is essential under stressful conditions. It has been reported that nutrient deprivation [31,32] and overfeeding [33,34], ketogenic and high carbohydrate diets [35,36], protein restriction [37,38,39], physical exercise [40,41,42], and other metabolic stresses, such as obesity, type 2 diabetes, or nonalcoholic fatty liver disease (NAFLD) [43], are capable of inducing FGF21 expression and/or signaling; and even more interesting, FGF21 metabolic effects will depend on the inducer factor or stress condition. FGF21 chronic treatment can induce fatty acid oxidation in WAT and suppress lipogenic genes in the liver to reduce triglyceride accumulation [44]. In other nutritional states, such as fasting, FGF21 can induce fatty acid oxidation and increase gluconeogenesis for glucose homeostasis maintenance [31,34]. Globally, FGF21 seems to be the key signal that communicates and coordinates the metabolic response to reverse different nutritional stresses and restores metabolic homeostasis. According the role of FGF21 under stress conditions, it is worth highlighting that the serum levels of this hormone are also induced under several pathogenic conditions. Hepatic production of FGF21 is increased under liver injury, viral infection, chemical insult, hepatosteatosis, steatohepatitis, NAFLD, cirrhosis, and liver cancer [43,45,46,47]. In skeletal muscle, besides its induction by insulin and exercise [40,48,49], FGF21 is also produced in mitochondrial myopathies [50,51] and it has been proposed as a putative serum biomarker for diagnostic use [52], but also in impaired mitochondrial fatty acid oxidation [53], muscle-specific autophagy deficiency [54], and transgenic overexpression of Akt1, perilipin-5 [55], or uncoupling protein 1 (UCP1) [56].

FGF21 may exert its effects by directly binding to its target tissues, such as adipose tissues, or through the central nervous system. Concretely, adipose tissues seem to be crucial for insulin-sensitizing actions of FGF21 or acute effects, but for energy expenditure induction, weight loss, or chronic effects, the central nervous system may be required [57]. 

**Figure 1:**
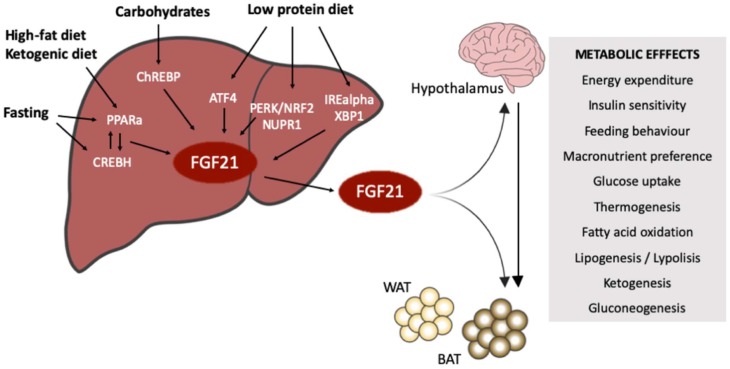
Fibroblast Growth Factor 21 (FGF21) induction by nutritional inputs. Low protein diet, carbohydrates intake, fat consumption, ketogenic diets and fasting are nutritional signals that induce the hepatic expression of FGF21 through different transcription factors. FGF21 as an endocrine factor exerts its metabolic effects mainly in the adipose tissues, directly or indirectly via a liver-hypothalamus axis. The metabolic effects of FGF21 look for restoring metabolic homeostasis by activating different metabolic pathways, but also by changing the macronutrient preferences and the feeding behavior.

To bring together the capacities of FGF21, this review is focused on describing individually the FGF21-dependent metabolic response activated by some of the most common nutritional challenges, the signal pathways implied, and the impact of these effects on global homeostasis (Figure 1). We consider this knowledge as essential for identifying the potential role of FGF21 in the onset and progression of some of the most prevalent metabolic pathologies, but even more important for understanding the potential of FGF21 as a target to counteract these diseases. 

## 2. FGF21 under Fasting-Refeeding Signaling

During the fasting state, several metabolic pathways are induced to maintain glycemia and energy homeostasis. Briefly, during early fasting, hepatic glycogenolysis is the main source of glucose. Later on, during long periods of fasting and essentially when most of the glycogen is depleted, hepatic gluconeogenesis and ketogenesis become the major energy sources. In this situation, WAT undergoes lipolysis to supply fatty acids and glycerol to the liver. Several hormones play an important role in regulating the metabolic adaptation to nutrient deprivation; molecules, such as insulin, glucagon, leptin, catecholamines, and more recently liver-derived FGF21, are well-known regulators of this metabolic state. In fact, FGF21 has been described as the missing link to fasting [28,34], and several authors have described its crosstalk with insulin and glucagon [58,59].

Several experiments have shown that, in mice, hepatic FGF21 expression is induced by 12 to 24 h of fasting [36,60,61]. FGF21 is then secreted from hepatocytes and activates ketogenesis, gluconeogenesis, lipolysis, fatty acid oxidation, and, globally, the metabolic adaptation to the fasting state [28]. In humans, no induction was seen up to 72 h of fasting [62]. The induction of FGF21 by fasting is only detectable after 7 to 10 days of nutrient deprivation and correlates with weight loss and the utilization of fuel derived from tissue breakdown [61,63]. Moreover, in humans, a high variation in the circulating levels of FGF21 during the fasted state has been detected [30]. 

FGF21 is a hepatokine deeply related to metabolic homeostasis maintenance, but how FGF21 exerts its metabolic effects is more complicated and not fully understood. The FGF21 mechanism of action includes direct effects on its target tissues and indirect effects through the central nervous system (CNS) [64]. It has been described that WAT, BAT, the heart, skeletal muscle, and the liver itself are FGF21 direct target organs/tissues [5,25]. Thus, FGF21 induces the peroxisome proliferator-activated receptor γ coactivator protein-1α (PGC1α), which regulates the gluconeogenic genes and increases the expression of glucose-6-phosphatase, phosphoenolpyruvate carboxykinase, carnitine palmitoyltransferase 1a, and hydroxymethylglutaryl-CoA synthase 2 [31,32,65]. By contrast, a brain-liver axis to maintain glucose homeostasis during prolonged fasting has been described [66]. It has been demonstrated that FGF21 can cross the blood-brain barrier, and peripheral organs FGF21, FGFR, and KLB are expressed in several regions of the brain too [67]. In this context, it has been proposed that FGF21 would exert its effects by inducing the ERK1/2 phosphorylation on the hypothalamic neurons, thereby stimulating the expression of the corticotropin-releasing hormone and finally activating the hypothalamic-pituitary-adrenal axis [66,68]. This activation underlies the activation of cyclic adenosine monophosphate (cAMP) response element-binding (CREB), which finally enhances the expression of glucogenic genes and sympathetic nerve activity in BAT [68,69].

FGF21 expression during the fasting state is driven mainly by the nuclear receptor peroxisome proliferator-activated receptor-α (PPARα) [32,36,60,61,70,71] and the cyclic adenosine monophosphate (cAMP)-responsive element-binding protein H (CREBH, encoded by CREB3L3) [72,73]. The promoter of FGF21 contains a peroxisome proliferator responsive element for PPARa and a CREBH binding site, indicating that FGF21 is under the dual control of both transcription factors [73,74,75,76]. Moreover, CREBH and PPARα are both cross-regulated by each other at the transcriptional level [77,78]. Finally, besides the role of CREBH in hepatic adaptation to energy starvation by the direct regulation of gene expression, CREBH also acts as a PPARα coactivator by participating in the recruitment of PGC1α to the FGF21 promoter [79]. 

To define more precisely how CREBH activates the FGF21 expression, some recent publications have associated the expression of CREBH and FGF21 with a change in the histone acetylation profile of the CREBH gene. Concretely, experiments with the MS-275, a class I-specific histone deacetylase (HDAC) inhibitor caused an increase in the histone H3 lysine 18 acetylation (H3K18ac), promote the hepatocyte nuclear factor 4α (HNF-4α) recruitment in the CREBH promoter, the CREBH expression, and finally the FGF21 production [80]. Furthermore, the CREBH activity, and by extension the FGF21 expression, is also regulated by the endoplasmic reticulum-associated protein degradation (ERAD) machinery concretely through the Sel1L-Hrd1 complex [81]. ERAD is responsible for recognizing and translocating protein for cytosolic proteasomal degradation. Expression is induced in the postprandial condition upon mouse refeeding. In this context, an inverse correlation between the Sel1L-Hrd1 complex and CREBH-FGF21 levels was described. The proposed mechanism is the Sel1-Hrd1 complex drives the polyubiquitination, turnover, and thus a reduction of nuclear CREBH in mice [82]. 

Other proteins that regulate FGF21 expression and function under nutrient deprivation are SIRT1, a NAD^+^-dependent deacetylase and Jumonji D3 (JMJD3), a histone demethylase. It is known that SIRT1 plays a critical role in mediating hepatic fasting responses through the deacetylation of different transcription factors or coactivators, such as PGC1α [83,84,85,86]. Regarding FGF21 induction, it has been described that under fasting conditions SIRT1 would be phosphorylated by cAMP/protein kinase A and thus would lead to the formation of a ternary complex with JMJD3 and PPARα [87]. This complex, apart from FGF21, would be responsible to induce genes related to mitochondrial b-oxidation, but no gluconeogenic genes [87]. 

Finally, it is worth mentioning that FGF21 also controls the fasting-refeeding transition. Some authors described that, during the refeeding period, there is an induction of the expression of the FGF21 in eWAT and a repression of fasting-induced FGF21 expression in the liver [88]. The authors used a temporal fasting-feeding cycle where mice had access to food for 4 h daily. Other authors, through liver-specific knockout mice, pointed out that the circulating FGF21 during the refeeding period would still be coming from the liver [34]. Although in this case the refeeding period also induced the expression of FGF21 in the eWAT, this induction did not have an impact in the serum levels. The mechanism underlying the eWAT induction of FGF21 is still unknown. In humans, the induction of plasma levels of FGF21 under increases of glucose and insulin levels was also observed [89,90]. These data highlight the pleiotropic role of FGF21 in the maintenance of metabolic homeostasis and its tissue and nutritional-specific regulation.

## 3. FGF21 and Carbohydrates

The carbohydrate content of the diet is able to modulate the hepatic expression of FGF21, mainly through the activation of the carbohydrate-responsive binding protein (ChREBP), and therefore its serum levels [91]. Interestingly, both the very-low carbohydrate diet [92,93] and the carbohydrate-rich diet [91,94] are able to induce the expression of FGF21. The maximum serum levels of FGF21 are found in low-protein high-carbohydrate intakes [95].

### 3.1. Ketogenic Diets (KDs)

Mice fed a ketogenic diet (KD) show significant increase in FGF21 expression and serum levels [96,97,98] required for the activation of hepatic lipid oxidation, triglyceride clearance, and ketogenesis induced by KD [36]. However, unlike the murine model, in obese patients, a KD does not increase FGF21 serum levels [99] and even decreases its levels when a KD is combined with low calorie intake [100]. Similarly, in obese children, a low carbohydrate diet (LCD) (50% carbohydrates) reduces FGF21 circulating levels [101].

In mice, a LCD also affects the WAT and BAT phenotypes due to FGF21 activity. In WAT, LCD reduces weight and decreases leptin secretion [92]. In addition, the high circulating levels of FGF21 cause an increase in UCP1 and CD137 mRNA levels [92], inducing browning in WAT [102]. In BAT, LCD also reduces weight and induces the mRNA expression of genes, such as FGF21, type 2 iodothyronine deiodinase (Dio2), or G protein-coupled receptor 120 (GPR120), which are correlated with BAT activation [92,103,104] or sensing dietary fat [105] and, therefore, in the control of energy balance in both humans and rodents. In concordance, KD induces protein UCP1 levels in this tissue [106].

While a LCD that increases FGF21 serum levels is able to ameliorate glucose and insulin tolerance in diabetic mice [98], a very-low carbohydrate diet fails to improve whole-body insulin resistance in rats [107]. In humans, the effect of LCD on hepatic or whole-body insulin resistance remains unclear [108]. However, a LCD is unable to improve NAFLD [92], while a long-term KD promotes lipid accumulation and hepatic steatosis in type 2 diabetes [96]. These data question the use of long-term very-low carbohydrate diets to lose weight [109].

### 3.2. High Carbohydrate Intake

Refeeding rats for 12 h with high-carbohydrate diets (HCDs) after a 24 h starvation period induces the mRNA expression of FGF21 in liver and increases its circulating levels [110]. In this case, the metabolic adaptations include an induction of lipogenesis, glucose uptake, glucose metabolism in the liver, a reduction of fatty acids uptake and fatty acid oxidation in the liver, induction of lipogenesis, glucose uptake, glucose metabolism, and lipolysis in WAT [110]. The same FGF21 induction is observed in mice fed a HCD containing 77% of energy as dextrose, 0.5% as fat, and 22.5% as protein [94]. 

In humans, overfeeding increases FGF21 levels [111]. However, carbohydrate overfeeding but no fat, led to marked increase of serum FGF21 in humans [89], and an acute response is found with a fructose load [35]. It appears that FGF21 increase is an attempt to maintain glucose homeostasis in a state of nutritional excess [89,112]. In this case, there are no discrepancies with murine models [113]. In mice, excess carbohydrate intake increases expression levels of UCP1, FGFR1c and KLB mRNA in BAT, suggesting increases in FGF21 sensitivity and energy expenditure. The expression of ChREBP mRNA in liver and BAT was increased in high-sucrose-fed mice. These results indicate that FGF21 participates in resistance to weight gain by a high-sucrose diet [113].

Finally, it has been described that in response to carbohydrate intake the hepatic production of FGF21 suppresses the sugar preference through a mechanism that involves hypothalamic signaling [114]. Hepatic FGF21 suppresses the consumption of simple sugars by acting on the paraventricular nucleus of the hypothalamus in a negative feedback loop. These data together with the GWAS studies available reinforce the role of FGF21 as a regulator of, at least, sugar and protein intake behavior [33,111,115]. 

## 4. FGF21 and Protein Intake

Amino acid starvation initiates a signal transduction cascade called Amino Acid Response. The first step is the activation of the general control nonderepressible 2 (GCN2) kinase. GCN2 2 is an amino acid sensor that, once activated, phosphorylates and inactivates the eukaryotic initiation factor 2a (eIF2a). The phosphorylation of the eIF2a factor leads to a repression of the global translation with the exception of some proteins that under the activating transcription factor 4 (ATF4) are produced to counteract the amino acid restriction [116]. ATF4 is part of the integrated stress response (ISR) in the liver and participates in the cellular response to different stressors, including amino acid deprivation. The metabolic effects of a low protein intake include an increase in food intake and energy expenditure and an alteration in amino acid, lipid and glucose metabolism [29,117]. Moreover, dietary amino acid composition and specific amino acids levels are also crucial for the activated metabolic response [29].

FGF21 is induced in mice not only by leucine, methionine/cysteine and asparagine deprivation but also by low-protein diets (LPD) [37,38,118,119] as a part of the transcriptional program mediated by ATF4 [37,39,120,121]. In humans, protein or specific amino acids-reduced diets also induce FGF21 levels [38,118,122,123]. Globally, FGF21 has been identified as a key mediator in the metabolic response associated with amino acid or protein intake deficiency [29,118]. No induction of FGF21 has been observed under caloric restriction when no protein reduction applies [124]. Under low protein intake, the induction of FGF21 mainly depends on the GCN2/ATF4 [120] pathway but GCN2-independent mechanisms have also been described [125,126]. These alternative mechanisms would include PERK [127] or other stress-related proteins such as the liver-integrated stress response–driven nuclear protein 1 (NUPR1) [38] or IREa-XBP1 [128]. PPARa signaling has also been implied in the induction of FGF21 under protein restriction [118].

It has been widely demonstrated that hepatic FGF21 is required for LPD-induced weight loss and increased energy expenditure [118]. It has been described that under amino acid deficient diets or LPD there is a reduction of de novo lipogenesis in the liver by inhibition of the fatty acid synthase (FASN), a reduction of lipogenic genes and an induction of lipolytic genes and finally an increased expression of thermogenic genes in BAT and WAT [37,39,129,130,131,132]. In this context, although most of the authors described the adipocytes as the targets for FGF21 action under LPD, a recent paper has included the central nervous system signaling as an intermediate step for the FGF21 metabolic response to low-protein diets [115]. Through deletion of the KLB co-receptor, Hill et al. [115] show that these mice are unable to switch on the metabolic response to a dietary protein restriction. 

The real question is that in LPD the caloric intake is usually achieved with an increment in the content of carbohydrates and it is sometimes difficult to distinguish between LPD or HCD. As has been mentioned before the expression of FGF21 is sensitive to nutrient deficiency and maximum serum levels of FGF21 are found in low-protein high carbohydrate (LPHC) intakes. In this regard, it has been published that in rats fed with a LPHC diet the increase of serum levels of FGF21 correlates with an increase in uncoupling protein 1 (UCP1), T-box transcription factor 1 and PRDM16 in perirenal adipose tissue (periWAT) [130]. This expression pattern, combined with the presence of multilocular adipocytes, suggested the occurrence of browning promoted by diet, a similar metabolic pattern observed with diets that are just defined as low-protein diets [130].

Instead of LPD for long periods, Li et al. [133] reported that the periodic LPHC diet shows similar metabolic benefits. Periodic LPHC causes an increase in FGF21 levels, an induction of the thermogenic program, and an obese-protected phenotype, despite an increased total energy intake. The problem with this diet is that the improvement in insulin sensitivity was lost within 14 days of switching back to the control diet, among which the FGF21 induction correlates with NUPR1 overexpression, suggesting a liver-integrated stress response [133].

## 5. FGF21 and Fat Consumption

The excess consumption of calorie-rich foods and sedentary lifestyle drive the actual global obesity epidemic and are the causes of several pathologic states, such as type 2 diabetes, cardiometabolic diseases, NAFLD, and the metabolic syndrome around the world. The impact of fat consumption on FGF21 expression and signaling is not easy to explain due to the variety of fat structures included in diets (long chain fatty acids saturated and unsaturated, medium-chain fatty acids, short chain fatty acids, fatty acids derivatives, and so on). 

Differences in FGF21 expression have been observed between mice fed a corn-oil high-fat diet versus mice fed a fish-derived long-chain polyunsaturated n–3 FA (PUFA)-enriched high-fat diet. The animals under corn-oil overfeed for five weeks showed more FGF21 mRNA levels than the ones whose intake was the above-mentioned PUFA [134]. In the same way, it has been described that FGF21 does not appear to be the major mechanism through which PUFA ameliorates high-fat diet (HFD)-associated metabolic disorders [134]. On the other hand, no changes in FGF21 expression have been detected between mice fed a high-fat diet (HFD) for 16 weeks and mice fed a low-fat diet [94]. In both approaches, soybean oil and lard were the sources of fat and caused the 60% Kcal for HFD and 10% Kcal in the low-fat diet. Significant differences have been described in neonatal mice where hepatic FGF21 expression is induced for suckling, probably due to the milk composition and its high fatty acids (FA) content [135]. In this case, the FGF21 secreted activates the thermogenic program in BAT. In humans, lipid infusion or a HFD overfeeding for five days increases the circulating levels of FGF21 [136,137,138]. By contrast, under a lipid tolerance test, a decrease in FGF21 levels has been described [139]. The induction of FGF21 expression in the liver by fatty acids undergoes through the activation of PPARα [24,135,136,140]. Some studies link the excess of fatty acid in obesity and type 2 diabetes with the overexpression of the FGF21 detected in these individuals [141,142].

It has been described that butyrate and α-lipoic acid also regulate the levels of FGF21 in the liver. Butyrate is mainly produced by intestinal microbiota through fermentation processes and is able to regulate gene expression due to its activity as a HDAC3 inhibitor [143]. Regarding α-lipoic acid, its dietary supplementation induces hepatic and plasma levels of FGF21 in vivo and in vitro [72,144,145] on a CREBH-dependent mechanism [72].

### FGF21 in Obesity: An Impairment in FGF21 Signaling

The excessive consumption of calories that exceed the storage capacity of the adipose tissue has been linked to low-grade inflammation, endoplasmic reticulum stress. and insulin resistance [146,147,148]. These defects increase the risk of metabolic diseases, such as nonalcoholic liver steatohepatitis (NASH), type 2 diabetes, cardiovascular diseases, and different forms of cancer [147,149]. FGF21 is considered an anti-obesity hormone, which circulates at variable levels and plays a role in mediating the physiological response to metabolic changes [150]. Some studies have shown that FGF21 metabolic benefits are mainly attributed to its activity in adipose tissue, where it induces thermogenic gene expression, oxygen consumption, and heat production [12,68,151].

Obesity is a state in which circulating levels of FGF21 are elevated in obese mice, in rhesus monkeys fed a HFD, and in the serum of overweight/obese humans [142,152,153,154,155]. This induction is probably a response to overcome excess energy income and triglyceride accumulation. However, in these situations, endogenous FGF21 levels appear to be ineffective, whereas high pharmacological doses, FGF21 analogues, agonistic antibodies, or small peptides targeting KLB induce its effects, promoting weight loss, improving glucose tolerance, and lowering serum free fatty acids [15,16,18,154,156,157,158,159,160]. This unresponsiveness to the endogenous FGF21 was firstly defined as an FGF21-resistant state [153]. However, the concept of FGF21 resistance is still controversial due to the undefined line between physiological and pharmacological effects of FGF21 and its mechanisms of action [154,161,162]. 

Some succeeding mechanistic research implied that FGF21 resistance is caused by a downregulation of its receptors, resulting in compensatory FGF21 production [153]. In this context, the published data suggest that the mRNA levels of the FGFR1c were reduced in the liver, WAT, and pancreas islets of obese mice [153]. Furthermore, KLB expression was also reduced in WAT and islets of obese mice [154,163]. FGFRs are widely expressed, but the KLB cofactor is present in only a small number of rodent tissues, notably adipose tissue, the liver, and the pancreas [20,21,164]. In mice with a total ablation of KLB, no FGF21 activity is detected. In those animals, the KLB disruption entirely abrogates acute FGF21 signaling in adipose tissue and liver [165]. In contrast, some new findings suggest that FGF21 activity is not mediated by a downregulation of KLB expression in WAT [166]. In an adipose tissue-specific KLB transgenic mouse, the maintenance of KLB protein expression in WAT does not alleviate the impairment in FGF21 signaling associated with obesity, thus indicating that the KLB expression downregulation may not be the major mechanism contributing to impaired FGF21 signaling in WAT [166]. FGF21 is also elevated in pathological conditions, such as obesity, insulin resistance, or liver diseases, and impairment in FGF21 signaling in these cases has also been described [43,141]. 

In humans, this resistance to FGF21 is not really understood, and two possible mechanisms have been proposed. The first mechanism implies the fibroblast activation protein (FAP), a serine protease that cleaves and inactivates the FGF21 [167,168]. The presence of FAP reduces the ratio of active FGF21 to total FGF21, limiting its metabolic effects even with the high circulating levels detected. It has been described that patients with type 2 diabetes have elevated levels of circulating FAP compared with nondiabetic subjects, thus suggesting that insulin resistance led to an inactivation of FGF21 and to the attenuation of its beneficial effects [90,169]. 

On the other hand, a common single-nucleotide polymorphism (SNP) in the KLB gene (rs2608819) has been identified and associated with a reduction in the levels of KLB in the adipose tissue and a higher body mass index (BMI) in obese subjects [163,170]. However, the expression of KLB, FGFR1, and FGFR3 in the liver are increased in obese humans, which may lead to an increased responsiveness of this tissue to FGF21. This hepatic overexpression may explain the effectiveness of a pharmacological administration of FGF21 or analogues. 

Further studies are needed to identify the ratio of inactive/active FGF21 in obese or diabetic individuals, because the problem may not be due to a FGF21-resistant state but a low amount of active FGF21. 

## 6. FGF21 and Exercise 

FGF21 gene expression in liver and serum levels are increased after acute exercise in mice and healthy humans (treadmill run or bicycle exercise at 50 to 80% VO2 max for 30 min in human subjects or treadmill run for 60 min in mice) or in individuals under intensive physical activity (two weeks program exercising at 85% of the maximal predicted heart rate for at least 15 min) [40,41,171]. The investigation of the exercise response in FGF21-deficient mice has shown that FGF21 action is necessary to achieve full metabolic benefits of exercise against the metabolic syndrome [172]. However, no changes in circulating FGF21 have been observed after eight weeks of endurance training in obese nondiabetic men [173] or after 10 weeks of either resistance or aerobic training in overweight women with type 2 diabetes [174]. In addition, the analysis of the effects of high-intensity interval training or moderate-intensity continuous training on cardiorespiratory fitness, body composition, blood glucose, and relevant systemic hormones in obese young women, shows significant improvements, despite the lack of training effects on body composition or significant systemic hormones, such as FGF21 [175].

Some methodological questions may explain these differences. For instance, different modes of exercise may be responsible; in fact, plasma FGF21 is reported to be increased with endurance exercise, but resistance training does not affect plasma concentration at any time [176]. In humans, there are no differences between serum levels of FGF21 before and immediately after the exercise; however, serum FGF21 levels are significantly higher than that before exercise after 1 h of recovery. In addition, after the 1 h recovery from high-intensity exercise (80% VO2 max), serum FGF21 levels are higher than that after the 1 h recovery from mild-intensity exercise (50% VO2 max) [40].

Coordinated regulation of glucagon and FGF21 by exercise has been established [176]. In humans, the liver secretion of FGF21 during exercise seems to be regulated by the glucagon to insulin ratio [177]. Accordingly, exercise-induced FGF21 secretion is impaired in patients with type 2 diabetes. In addition, the exercise-induced augmentation in plasma FGF21 is attenuated by a pancreatic clamp, blocking the increase in the glucagon to insulin ratio in healthy individuals, confirming the role of these pancreatic hormones as upstream regulators [42]. The same authors also described the putative role of free fatty acids (FFA) as a regulator of liver FGF21 gene expression through the activation of the nuclear receptor PPARα, the exercise induction of serum FFA, and the hampering of this response by the pancreatic clamp. Increased FGF21 gene expression in the liver with acute exercise is accompanied by elevated gene expression, not only of PPARα, but also of ATF4 [40]. These data point to a synergistic effect of glucagon and FFA regulating the expression of FGF21 in the liver during exercise. Accordingly, a study with elderly Japanese men revealed a reduction of serum FGF21 levels mediated by reduction of hepatic fat content after a five-week endurance exercise program [178]. They also suggest that endurance exercise modulates hepatic fat content and FGF21 resistance, regardless of obesity status [178]. From the results obtained in mice studies, it has been proposed that exercise may induce the adipose expression of the FGF21 receptors, FGFR1, and KLB, via PPARγ-mediated transcriptional activation. Thus, in mice, exercise would sensitize adipose tissue to the FGF21 actions, thus promoting beneficial effects on glucose metabolism [179,180]. 

## 7. Concluding Remarks: Is FGF21 a Therapeutic Target for Obesity and Insulin Resistance in Humans?

Besides its physiological induction, FGF21 is also elevated in pathological conditions, such as obesity, insulin resistance, or liver diseases, and impairment in FGF21 signaling in these cases has also been also described [43,141,181]. Many questions are still open regarding the metabolic role of FGF21 in humans, especially under nonpathological conditions.

Some divergent data between mice and humans have been detected regarding the mechanisms that induce the FGF21 expression, but also on its metabolic effects. As has been mentioned before in this review, neither short-term fasting nor KD increase FGF21 serum levels in prolonged fasting (7–10 days). By contrast, in humans, it seems that FGF21 is secreted under carbohydrate intake as a postprandial hormone produced under the insulin signal [35,89,90,137], protein intake [39,118], or exercise [182]. A positive correlation between insulin and FGF21 levels has been described [142,163], and a genetic variant of FGF21 has been associated with an increased sugar intake [183]. Genetic studies in humans have associated some SNPs in and around the FGF21 gene with carbohydrates, protein, fat, and alcohol preferences [33,184,185], but also a specific KLB expression profile has been described in humans. Beyond the liver and adipose tissue, in humans, KLB is also detected in the breast, bone marrow, and pancreas [186]. These differences may explain, at least in part, the particular metabolic effects of FGF21 in humans.

Initially, FGF21 was described as a promising target to treat obesity and insulin resistance. At present, the approaches to design FGF21 analogues to use as antidiabetic drugs have not reached the expected results. FGF21 analogues tested in overweight/obese patients with T2D are able to reduce dyslipidemia and steatosis, but no improvement in glycemic and body weight was obtained [8]. Recent data in humans clearly imply FGF21 in dietary preferences, appetite, and lipid profile, but also described KLB as a potential regulator of FGF21 action and FAP as a putative target to control FGF21 activity. 

Altogether, let us hypothesize that FGF21 can be a target to treat obesity and several metabolic disorders, but perhaps not in the way that was previously proposed. More research is needed to understand the mechanisms underlying the role of FGF21 in macronutrient preference and food intake behavior, KLB regulation, and FAP activity to uncover FGF21 signaling as a potential therapeutic approach. In humans, a successful therapeutic approach that focuses on designing specific inhibitors of FAP to overcome the FGF21-resistant state associated with obesity and type 2 diabetes would succeed. 

## Abbreviations

ATF4	activating transcription factor 4
BAT	brown adipose tissue
ChREBP	carbohydrate responsive binding protein
CNS	linear dichroism central nervous system
CREBH	cyclic adenosine monophosphate (cAMP)-responsive element-binding protein H
Dio2	type 2 iodothyronine deiodinase
eFGF	Endocrine FGFs
eIF2A	eukaryotic initiation factor 2 alpha
ERAD	endoplasmic reticulum-associated protein degradation
FFA	free fatty acids
FAP	fibroblast activation protein
FASN	fatty acid synthase
FFA	free fatty acids
FGF21	Fibroblast Growth Factor 21
FGFR	Fibroblast growth factor tyrosine kinase receptor
FRS2a	FGFR substrate 2a
GCN2	general control nonderepressible 2
GPR120	G protein-coupled receptor 120
HCD	high carbohydrate diet
HDAC	histone deacetylase
HFD	high-fat diet
HNF4	hepatocyte nuclear factor 4 alpha
JMJD3	Jumonji D3
KD	ketogenic diet
KLB	beta-klotho
LCD	low carbohydrate diet
LPD	low-protein diet
LPHC	low-protein and high carbohydrate
NAFLD	nonalcoholic fatty liver disease
NASH	nonalcoholic liver steatohepatitis
NUPR1	liver-integrated stress response–driven nuclear protein 1
PGC1a	peroxisome proliferator-activated gamma coactivator 1 alpha
PPARα	Peroxisome proliferator-activated receptor alpha
PPARγ	Peroxisome proliferator-activated receptor gamma
TBX1	T-box transcription factor 1
UCP1	uncoupling protein 1
WAT	white adipose tissue
